# Innovative Acrylic Resin-Hydrogel Double-Layer Coating: Achieving Dual-Anchoring, Enhanced Adhesion, and Superior Anti-Biofouling Properties for Marine Applications

**DOI:** 10.3390/gels10050320

**Published:** 2024-05-07

**Authors:** Boning Jiang, Yuhan Zhang, Ruiyang Wang, Ting Wang, En Zeng

**Affiliations:** 1 Aulin College, Northeastern Forestry University, Harbin 150040, China; boningj@tom.com (B.J.); zhang18846363161@nefu.edu.cn (Y.Z.); wangry_sunny@nefu.edu.cn (R.W.); 2 College of Chemistry, Chemical Engineering and Resource Utilization, Northeast Forestry University, 26 Hexing Road, Harbin 150040, China; 3 Rongbang Chemical Co., Ltd., Suining 629000, China

**Keywords:** hydrogel coating, gel microspheres, adhesion strength, marine anti-biofouling, cuprous oxide

## Abstract

Traditional anti-corrosion and anti-fouling coatings struggle against the harsh marine environment. Our study tackled this by introducing a novel dual-layer hydrogel (A-H DL) coating system. This system combined a Cu_2_O–SiO_2_–acrylic resin primer for anchoring and controlled copper ion release with a dissipative double-network double-anchored hydrogel (DNDAH) boasting superior mechanical strength and anti-biofouling performance. An acrylamide monomer was copolymerized and cross-linked with a coupling agent to form the first irreversible network and first anchoring, providing the DNDAH coating with mechanical strength and structural stability. Alginate gel microspheres (AGMs) grafted with the same coupling agent formed the second reversible network and second anchoring, while coordinating with Cu^2+^ released from the primer to form a system buffering Cu^2+^ release, enabling long-term antibacterial protection and self-healing capabilities. FTIR, SEM, TEM, and elemental analyses confirmed the composition, morphology, and copper distribution within the A-H DL coating. A marine simulation experiment demonstrated exceptional stability and anti-fouling efficacy. This unique combination of features makes A-H DL a promising solution for diverse marine applications, from ship hulls to aquaculture equipment.

## 1. Introduction

The maritime water environment presents significant challenges, including chemical corrosion and biological fouling, prompting intensified efforts to develop effective anti-corrosion and anti-fouling solutions for marine vessels [1,2,3]. The traditional coatings used in shipbuilding exhibit inherent limitations, such as a susceptibility to microbial adhesion, a rapid depletion of anti-fouling agents [4], a restricted lifespan [5], and environmental concerns [6], underscoring the need for innovative approaches. 

Nowadays, the research on emerging anti-fouling technologies or coatings is forced in two directions. The first one is replacing the traditional coatings with more environmentally friendly fungicides [7], and the second one involves biocide-free coatings that exploit surface chemistry to reduce adhesion or dislodge adhered organisms [8]. Both of those strategies can solve one aspect of the challenges mentioned above. However, they all have some limitations [1]. For instance, these more environmentally friendly fungicides, such as degradable synthetic organic or natural product-based biocides, have a lower efficiency than the traditional ones, such as copper-based coatings, and the biocide-free coatings with a special surface cannot completely restrain bio-fouling and ensure the mechanical performance.

In response to these challenges, hydrogel coatings have emerged as a promising solution [9,10,11]. Characterized by an intricate network of interwoven water molecules and polymer chains, hydrogel coatings demonstrate an exceptional durability [12,13]. Specifically, within the realm of marine hydrogel coatings, the surface microstructure of gel coatings proves less conducive to microorganism adhesion, resulting in an extended service life. Moreover, the smooth surface of hydrogels reduces resistance during a ship’s forward movement [14]. This combination of attributes positions hydrogel coatings as a promising avenue for tackling the challenges associated with marine environments.

In the realm of hydrogel coating research, pivotal attention is directed toward two key areas: the mechanical properties of the hydrogel itself and its interfacial toughness with the substrate. For the mechanical properties of the hydrogel, its performance is a critical benchmark for engineering applications [12]. Among the existing studies, double-network hydrogels have emerged as a common choice for enhancing these mechanical properties. These hydrogels boast exceptional tensile [15] and impact resistance owing to their unique dual-network structure. In current research, dual-network hydrogels are typically fashioned by combining a dissipative network and a solid network [16]. The solid network, primarily cross-linked irreversibly, provides the hydrogel with significant mechanical strength [9,16,17,18]. Conversely, the dissipative network, typically cross-linked reversibly, dissipates external stress by undergoing the fracture and regeneration of its cross-linking nodes, thereby fortifying the hydrogel’s toughness and refining its mechanical characteristics.

Considering the application scenarios of hydrogel marine coatings, dual-network hydrogels prove particularly well-suited [16,19,20,21,22,23]. They ensure the stability of the gel coatings over extended periods, even under diverse underwater conditions, thereby offering prolonged protection to the substrate [24]. Turning to interfacial toughness, hydrogel materials have remained a focal point across various application domains since their inception [9]. The work of Yuk et al., researchers who achieved groundbreaking progress by covalently anchoring hydrogels to non-porous surfaces and elastomers, introduced a paradigm shift in hydrogel coating research and development [24,25]. On the basis of this research, hydrogel coating technology in shipbuilding has seen rapid advancement in recent years, with various structures demonstrating excellent underwater anti-fouling performance.

However, challenges persist, particularly regarding the covalent anchoring strategy. While this method has shown promise, its applicability is limited to substrates with active hydroxyl groups on their surfaces, such as glass and ceramics [26,27]. For materials commonly used in ships, such as aluminum and steel, surface plasma treatment is required to impart anchoring activities [28,29], which is costly and not feasible for industrial applications. Moreover, the soft nature of hydrogel materials renders them susceptible to damage in the unpredictable ocean environment, exposing substrates to corrosion [9,12]. In addition, efforts to incorporate antibacterial agents, such as Cu^2+^, into hydrogels for functional antibacterial effects face challenges, but the antibacterial agents tend to leach into seawater at an accelerated rate, diminishing the functional lifespan of the hydrogel coating.

Building upon the aforementioned research on hydrogel marine coatings, this study introduces an innovative acrylic resin–hydrogel double-layer (A-H DL) marine anti-corrosion and anti-fouling coating structure. The A-H DL coating comprises an acrylic resin (AR) primer, which includes polyvinylpyrrolidone-cuprous oxide (PVP-Cu_2_O) microcapsules and 3-(trimethoxymethylsilyl) propyl methacrylate-modified nano-silica (TMAPMS-SiO_2_), and a dissipative double-network double-anchored hydrogel (DNDAH) coating, showcasing commendable mechanical properties, robust adhesion, and effective anti-biosorption functionality. This double-layer hydrogel coating structure addresses the challenges associated with the low-cost anchoring and long-term underwater anti-biosorption of hydrogel coatings by incorporating a primer coating between the gel layer and the substrate. The primer is designed to provide anchoring active sites and facilitate copper ion release, enhancing antibacterial performance. Additionally, in the event of gel coating damage, the primer serves as a final protective barrier. The A-H DL coating, which boldly uses a hydrogel surface to achieve chemical structure anti-fouling, adheres to the design concept of environmental protection of modern marine coatings while retaining the efficient anti-biofouling performance of traditional copper-based coatings, and it also improves its mechanical performance through innovation, thus achieving dual-anchoring, enhanced adhesion, and superior anti-biofouling properties for marine applications.

## 2. Result and Discussion

### 2.1. Construction of the A-H DL Coating System

**Figure 1:**
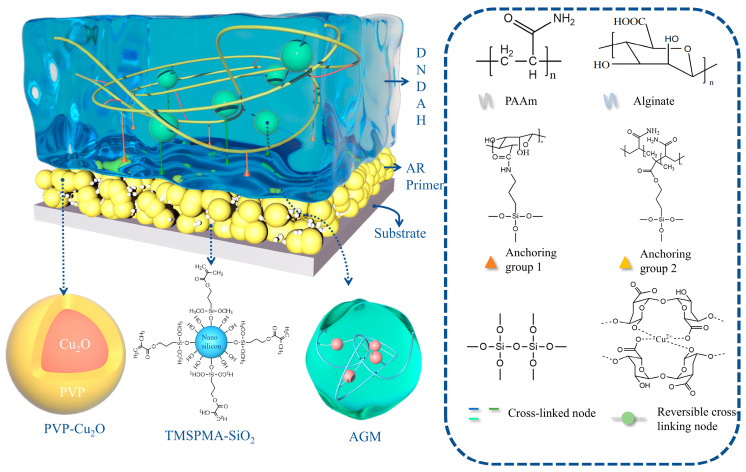
The basic structure of the A-H DL coating.

The coating system of A-H DL combined a Cu_2_O-SiO_2_-AR primer for anchoring and controlled copper release with a mechanically strong and anti-fouling DNDAH. The detailed internal structure of the coating is depicted in Figure 1.

For the primer system, an acrylic resin coating served as the foundation, with the addition of Cu_2_O particles coated with PVP to impart an antibacterial functionality. Incorporating TMSPMA-modified nano-silica particles enhanced the anchoring ability of the primer and gel system.

For the hydrogel component, we adopted the construction strategy of a double-network hydrogel. The AAm monomer was copolymerized and cross-linked with a coupling agent to form the first network, with the coupling agent condensing to create irreversible cross-links. The second network incorporated AGM grafted with a coupling agent. Alginate coordinated with the Cu^2+^ released from the Cu_2_O in the primer, forming reversible cross-links. The Cu^2+^ was retained in the gel for an extended period, ensuring long-term antibacterial protection for the hull. The coating’s outstanding mechanical properties and prolonged anti-biosorption function were experimentally verified. The two networks interconnected through the condensation of the coupling agents, and the anchoring groups on both networks’ coupling agents underwent condensation with the silicon hydroxyl groups of the SiO_2_ in the primer, forming covalent anchoring with verified super-anchoring performance to form the A-H DL coating. The chemical composition, excellent mechanical properties, and marine anti-fouling functionality of the A-H DL coating were systematically demonstrated through subsequent characterization and performance experimental test results.

### 2.2. Analysis of the Internal Chemical Structure of the Coatings

#### 2.2.1. Internal Structure of DNDAH

The hydrogel coating in this study consisted of a robust first network, formed by the copolymerization and cross-linking of a coupling agent (TMSPMA) and acrylamide, and a dissipative second network created by the physical cross-linking of copper and a long chain of alginate (Cu^2+^ gradually released from the primer), modified by a coupling agent (APES).

**Figure 2:**
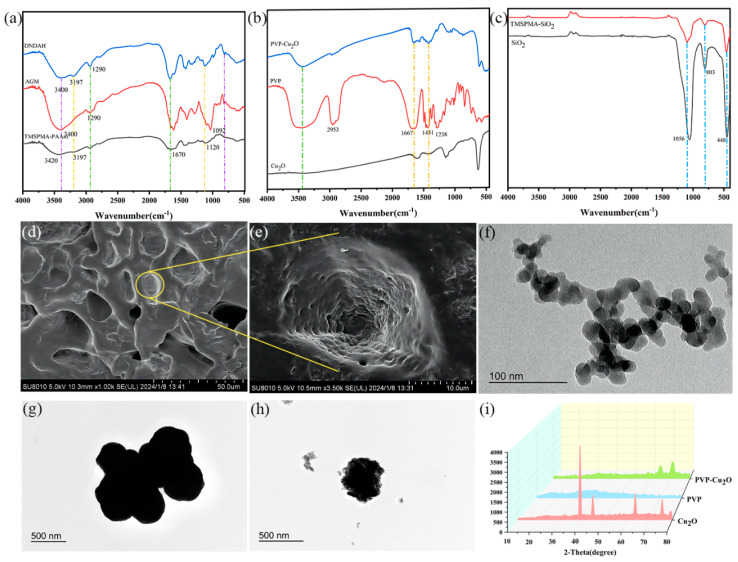
Chemical composition and morphology characterization results of the A−H DL coating. FTIR spectra of (**a**) hydrogel, (**b**) Cu_2_O, PVP-Cu_2_O, and PVP, and (**c**) SiO_2_ and TMSPMA-SiO_2_. SEM image of (**d**) surface morphology of DNDAH and (**e**) surface morphology of the gel microsphere. TEM image of (**f**) TMSPMA-SiO_2_, (**g**) Cu_2_O, (**h**) PVP-Cu_2_O, and (**i**) XRD patterns of Cu_2_O, PVP, and PVP-Cu_2_O.

Firstly, we conducted an FTIR analysis to identify the functional groups in the hydrogel samples. The results for TMSPMA-PAAm, AGM, and DNDAH are presented in Figure 2a.

In the TMSPMA-PAAm sample spectrum, the broad absorption peak at 3420 cm^−1^ corresponded to the silanol (Si–OH) stretching vibration, while 3197 cm^−1^ was the characteristic absorption peak of the associated –NH_2_. Additionally, the peak at 1120 cm^−1^ corresponded to the Si–O–Si bond’s antisymmetric stretching vibration, and the peak at 1670 cm^−1^ corresponded to the stretching vibration of the C=O bond in amides. Notably, there was no absorption peak for the terminal ene groups. This infrared spectrum confirmed the successful copolymerization of the AAm monomer with TMSPMA, with some coupling agents undergoing condensation reactions to cross-link into a hydrogel, while others retained the silicon hydroxyl groups as the anchor points.

In the AGM sample spectrum, a similar backbone with TMSPMA-AAm was observed. A broad absorption peak at 3400 cm^−1^ was attributed to the silanol (Si–OH) stretching vibration, and the adsorption peak at 1670 cm^−1^ corresponded to the stretching vibration of the C=O bond in amides. Additionally, 1290 cm^−1^ represented the ether bond absorption peak in the long chain of alginate, while the peak at 1092 cm^−1^ corresponded to the Si–O–Si bond’s antisymmetric stretching vibration. Importantly, there was no absorption peak for the carboxyl (–COOH) and primary amine (–NH_2_) groups. The infrared spectrum results indicated the successful grafting of the coupling agent onto the main chain of alginate, with some coupling agents cross-linked into hydrogels through condensation reactions, while others retained the silyl hydroxyl group as an anchor point and cross-linking site with the first network.

On the other hand, the spectrum of the DNDAH sample combined the infrared spectrum characteristics of the TMSPMA-AAm and AGM samples. It exhibited an absorption peak at 3400 cm^−1^ corresponding to alcohol hydroxyl, originating from the two types of networks. The peak at 3197 cm^−1^ represented the characteristic absorption peak of the associated –NH_2_, derived from the primary amine in the TMSPMA-AAm network. Additionally, the peak at 1290 cm^−1^ corresponded to the ether bond’s absorption peak, consistent with the spectrum of AGM. Therefore, it was preliminarily inferred that the DNDAH sample comprised both TMSPMA-AAm and AGM networks, demonstrating an anchoring performance.

Furthermore, the SEM images of the hydrogel sample (Figure 2d,e) showed the 3D network structure of the hydrogel to a certain extent. The water that initially occupied most of the space in the hydrogel was removed by freeze-drying, leaving only the polymer skeleton, which retained the original hollow shape to a certain extent. Firstly, Figure 2d illustrates that the internal structure of the polymer is porous, aligning with the internal structural characteristics of the hydrogel [30]. Additionally, the polymer exhibits a high degree of cross-linking, and research indicates that the higher the cross-linking degree of a material, the better its anti-swelling performance. Therefore, the low swelling rate of the hydrogel coating can be explained in terms of the 3D structure, which is conducive to the long-term adhesion of the hydrogel coating onto the primer surface. Secondly, in Figure 2e, the presence of smaller spherical particles in the network structure is apparent. Upon zooming in, Figure 2e shows microspheres with a diameter of approximately 20 μm. According to the theoretical gel structure, it can be inferred that these are coupling agent-cross-linked alginate gel microspheres dispersed in the hydrogel system, confirming the theoretical structure of the hydrogel’s double network.

#### 2.2.2. Internal Structure of AR Primer

The AR primer contained acrylic resin and some additives that helped the coating solidify, as well as nano-PVP-Cu_2_O particles and modified nano-SiO_2_ dispersed in the coating. The following mainly displayed the chemical structure and morphological characteristics of the two types of nano-particles.

On the one hand, for the PVP-Cu_2_O sample, we characterized its chemical composition and morphological characteristics. Firstly, we conducted an FTIR analysis to verify the composition and embedding integrity of the PVP-Cu_2_O microcapsules. The results of Cu_2_O, PVP, and PVP-Cu_2_O are shown in Figure 2b. Characteristic absorption peaks at 2953 cm^−1^ and 1431 cm^−1^ (–CH), 1667 cm^−1^ (C–O), and 1283 cm^−1^ (C–N) were found. The absorption peak at 3470 cm^−1^ was the characteristic peak of –OH, which was supposed to be derived from the water adsorbed by the PVP. For the spectrum of Cu_2_O, the absorption peak at 628 cm^−1^ corresponded to Cu_2_O, and the absorption peak at 3310 cm^−1^ was supposed to be caused by the moisture in the tested sample. For the spectrum of PVP-Cu_2_O, the characteristic absorption peak of Cu_2_O was found at 607 cm^−1^. Moreover, characteristic absorption peaks at 2971 cm^−1^ and 1416 cm^−1^ (–CH) and 1653 cm^−1^ (C–O) were also found. These results show that the components of the produced microcapsules were PVP and Cu_2_O [31]. In addition, we further validated the chemical composition of the PVP-Cu_2_O microcapsules by XRD. Figure 2i shows the XRD profiles of Cu_2_O, PVP, and PVP-Cu_2_O, respectively. It can be observed from the XRD pattern of Cu_2_O that six major characteristic X-ray diffraction peaks appeared at positions 2θ of 31.3°, 38.1°, 44.1°, 63.2°, 75.5°, and 79.4°, whereas PVP, as an organic noncrystallographic material, had no obvious crystal characteristic peaks in the XRD pattern. For the PVP-Cu_2_O XRD pattern, the characteristic diffraction peaks of 38.1° and 44.1° still existed, but the intensity was greatly reduced, and the remaining weaker characteristic peaks were intermixed with the hybrid peaks of PVP, indicating that the PVP phase interfered with the X-ray diffraction intensity of Cu_2_O, further proving the presence of Cu_2_O and PVP in PVP-Cu_2_O.

We could observe the specific morphological characteristics and particle size of the microcapsules through TEM. Figure 2f,g show the Cu_2_O particles without PVP and the microcapsules with PVP, respectively. Firstly, these microcapsules have a spherical shape in appearance and a distinct core–shell structure, with a spherical radius of approximately 200 nm and a shell thickness of approximately 40 nm, proving the inclusion structure of PVP-Cu_2_O.

Then, we also characterized the chemical composition and morphological characteristics of the TMSPMA-SiO_2_ sample. First of all, we performed an FTIR analysis to explore the influence of coupling agent modification on the chemical structure of nano-SiO_2_. The results of SiO_2_ and TMSPMA-SiO_2_ are shown in Figure 2c. For the characteristic absorption peak of Si–OH at 1056 cm^−1^, the absorption of TMSPMA-SiO_2_ was significantly weaker than that of the unmodified SiO_2_, indicating that the modification of the coupling agent reduced the hydroxyl’s density on the silica surface, thus weakening the agglomeration of silica during the high-temperature curing of the paint film. Moreover, we observed the morphology and particle size of TMSPMA-SiO_2_ through TEM. Figure 2h shows that the particle size of TMSPMA-SiO_2_ was around 50 nm.

### 2.3. Anchoring Performance and Mechanical Properties of A-H DL Coating

#### 2.3.1. Internal Tensile Strength of DNDAH

**Figure 3:**
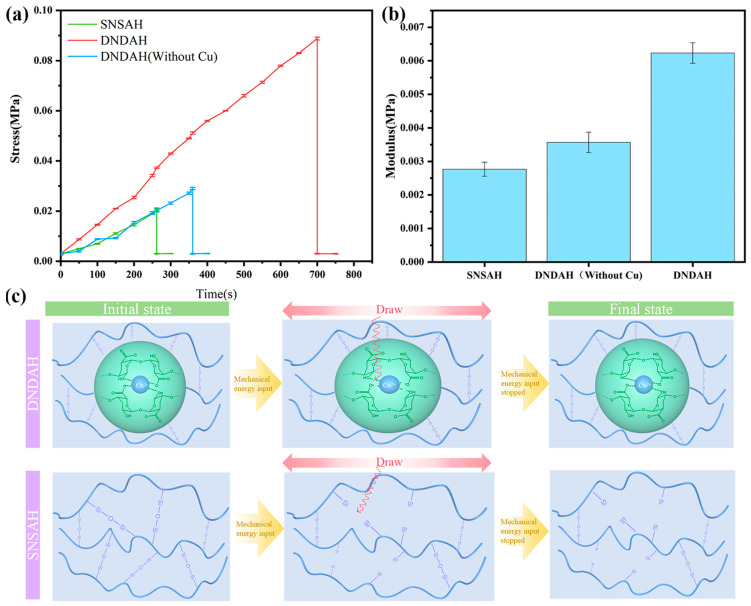
DNDAH tensile performance. (**a**) Tensile strength of DNDAH, SNSAH, and DNDAH without Cu^2+^. (**b**) The modulus of the extension of DNDAH, SNSAH, and DNDAH without copper. (**c**) DNDAH dissipation mechanism.

The tensile strength is one of the crucial indicators of mechanical properties [32]. We conducted tensile strength tests on SNSAH and DNDAH at room temperature and obtained Figure 3. The tensile properties of SNSAH were significantly weaker than those of DNDAH.

According to the calculation results, the calculated tensile strength of SNSAH was 0.020 MPa, and the tensile strength of DNDAH without Cu^2+^ was 0.29 MPa, which was the same as that of SNSAH, whereas that of DNDAH was 0.073 MPa (Figure 3a). From Figure 3b, we can observe that the modulus of DNDAH was obviously higher than those of SNSAH and DNDAH without Cu^2+^. This verified the dissipation effect of copper AGM on external stress. This tensile strength was similar to the tensile strength of interpenetrating double-network hydrogels reported in previous studies [33,34], indicating that the mechanical properties and dissipative properties of the dissipative gel microspheres and those of an interpenetrating dissipative gel are the same, but AGM can provide a second anchoring effect on the primer. Figure 3c demonstrates the mechanism of how DNDAH dissipates external mechanical energy.

#### 2.3.2. The Adhesion Properties of A-H DL

**Figure 4:**
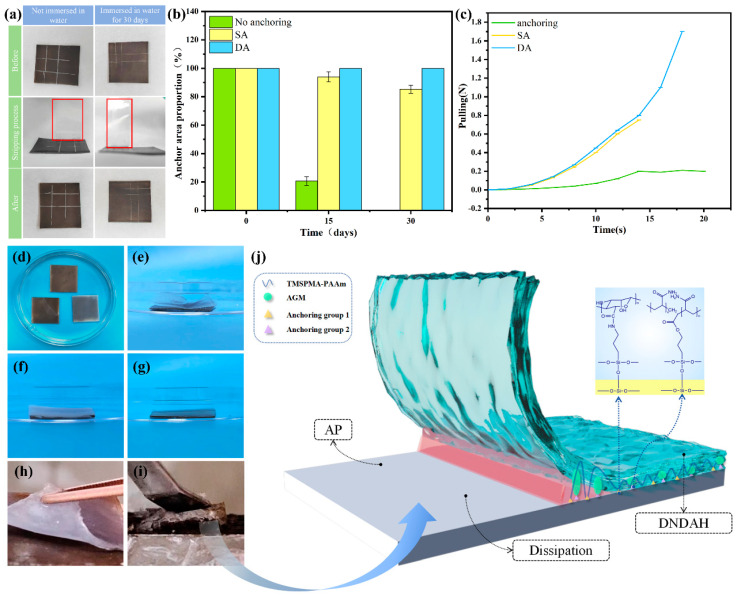
DNDAH anchoring performance. (**a**) The results of the cross-cut test. (**b**) The adhesion area of the DNDAH on the AR primer and long−term underwater anchoring performance. (**c**) Peeling force–time curve. (**d**) Bird’s-eye view of (**e**–**g**): (**e**) the paint coat composed of the primer without TMSPMA-SiO_2_ combined with DNDAH; (**f**) the paint coat composed of the primer with TMSPMA-SiO_2_ combined with AAm single-anchoring hydrogel; and (**g**) the paint coat composed of the primer with TMSPMA-SiO_2_ combined with DNDAH. (**h**) Unanchored hydrogel coating’s peeling. (**i**) Anchored hydrogel coating’s stripping. (**j**) Anchored coating’s peeling dissipation mechanism.

The adhesion properties of the product included the anchoring properties between the DNDAH and the AR primer and the adhesion between the AR primer and the substrate. All the results of the experiments are displayed in Figure 4.

Firstly, we proved the excellent adhesion properties between the AR primer and the substrate via a cross-cut test. The results of the experiment are demonstrated in Figure 4a. We found that almost no paint film was peeled off from the substrate by the tape (the tape marked by the red box is very clean). Then, the AR primer that had been immersed in water for 30 days showed a similar experiment phenomenon as the primer mentioned above. Hence, the AR primer had great adhesion properties to the substrate.

Furthermore, we verified the excellent anchoring performance between the DNDAH and the AR primer coating through an underwater swelling and detachment experiment and peeling experiments. The anchoring structure was formed by the condensation of the hydroxyl (–OH) provided by TMSPMA-SiO_2_ on the AR primer surface with two silane coupling agents in the gel system. The interface adhesion and dissipation mechanisms of the SA hydrogel are shown in Figure 4j.

In the underwater swelling and detachment experiment, shown in Figure 4b, there was a change in the area percentage of the hydrogel anchored to the primer over time. It was clear that the percentage of the hydrogel with double-anchoring (DA) remained constant over 30 days, while the percentage of the single-anchoring (SA) hydrogel suffered a moderate decrease. However, the hydrogel without anchoring detached completely. The anchoring of the hydrogel coating and the primer coating after immersion in water for 30 days is shown in Figure 4d–g. Figure 4e shows the paint coat composed of the primer without TMSPMA-SiO_2_ combined with DNDAH; Figure 4f shows the paint coat composed of the AR primer with TMSPMA-SiO_2_ combined with SNSAH; Figure 4g illustrates the AR primer composed of the primer with TMSPMA-SiO_2_ combined with DNDAH. Upon comparing the results shown in Figure 4e,g, it could be observed that the DNDAH coating on the primer without TMSPMA-SiO_2_ had almost completely detached. However, the DNDAH coating on the primer with added TMSPMA-SiO_2_ exhibited no edge warping or bulging. This phenomenon proved that TMSPMA-SiO_2_ provided the Si-OH capable of undergoing a condensation reaction with the coupling agent in the DNDAH coating, forming a chemically anchored interface between the two coatings and the primer. Additionally, upon comparing the results in Figure 4f,g, it was evident that SNSAH displayed partial edge warping, while the double-anchored hydrogel did not show any edge warping. This phenomenon indicated that the DNDAH had a more robust and enduring anchoring effect on the AR primer when TMSPMA-SiO_2_ was added.

In the peeling experiment, the unanchored sample could peel off the hydrogel coating from the AR primer with only 0.2 N, while the SA hydrogel and DA hydrogel failed to peel off the hydrogel coating from the AR primer, until it broke, indicating that the interfacial toughness between the hydrogel coating and the AR primer was greater than the internal toughness of the hydrogel (Figure 4c). The interface adhesion performances of the peeling DA and SA hydrogels are illustrated separately in Figure 4h,i. We can observe that some part of the DA hydrogel remained on the AR primer, while the SA hydrogel could be peeled off successfully.

To sum up, the two layers of the A-H DL coating could be connected extremely firmly via the covalent anchoring structure, and the entire coating had great adhesion properties with respect to the substrate. Hence, the brilliant adhesion of the A-H DL coating could be proven.

### 2.4. Functional Evaluation of the Coatings

#### 2.4.1. Cu^2+^ Release Performance

**Figure 5:**
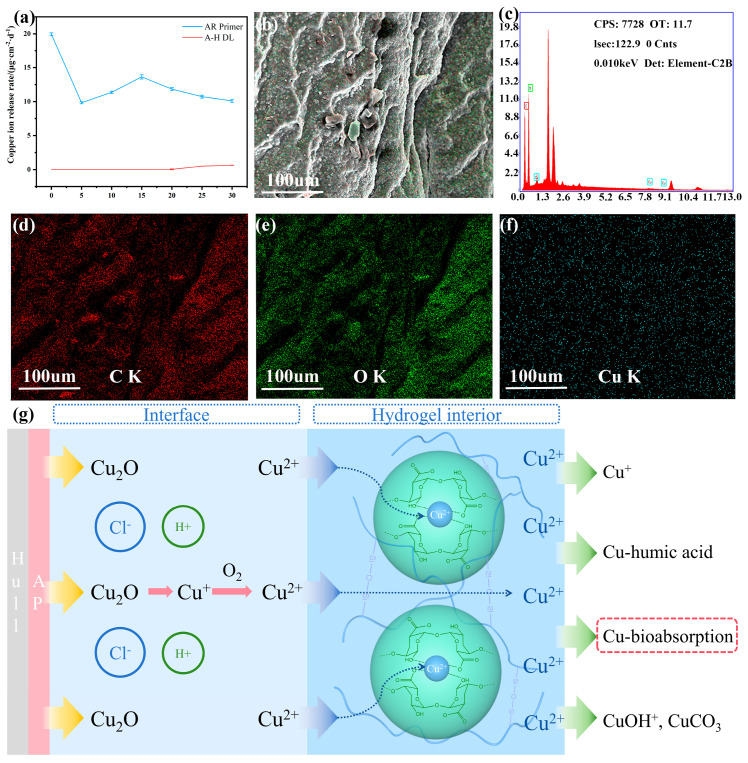
The Cu^2+^ release performance of the AR primer and the A-H DL coating. (**a**) Copper ion release rate curves of the samples (AR primer and A-H DL coating). Elemental analysis of the DNDAH coating after the slow release of Cu^2+^ in (**b**) mapped areas and (**c**) elemental distribution. (**d**) Elemental mapping of C. (**e**) Elemental mapping of O. (**f**) Elemental mapping of Cu. (**g**) Cu^2+^ release mechanism.

The results of the Cu^2+^ release properties’ experiment are demonstrated in Figure 5a–c. Regarding the results of the determination of the Cu^2+^ release rate (Figure 5a), the initial copper ion release rate of the sample AR primer reached up to 20 μg·cm^−2^·d^−1^. Then, there was a significant decline, during which the rate dropped to 9.8 μg·cm^−2^·d^−1^ on the fifth day. Subsequently, after rising to 13.6 μg·cm^−2^·d^−1^ on the fifteenth day, the rate dropped again and tended to stabilize gradually. It could be deduced that, due to the presence of a large amount of Cu_2_O in the coating, the release rate of the copper ions was very fast in the initial stage. Then, the Cu_2_O value in the surface layer of the coating rapidly decreased, resulting in a significant decrease in the release rate. As the soaking time progressed, the internal structure of the coating became loose, intensifying the release of copper oxide and increasing the release rate of the copper ions to the maximum value. Finally, with the consumption of Cu_2_O, the release rate gradually decreased and eventually stabilized [35]. The behavior of the AR primer releasing Cu^2+^ into the artificial seawater was similar to that of the AR primer releasing Cu^2+^ into DNDAH because of the highwater content of the hydrogel. This release pattern ensured that the Cu^2+^ could release into the hydrogel immediately to form the reversible cross-linking structure. Meanwhile, the release rates of A-H DL were always zero before 20 days and were still very small after that time. This phenomenon indicated that DNDAH provided a good buffering effect on the release process of Cu^2+^, thus decreasing the pollution of Cu^2+^ in the marine environment. 

Figure 5b shows the mapped scan area. Figure 5c displays the elemental content of the samples. Figure 5d,e indicate that there are many C and O elements in DNDAH and further prove that the skeleton of this hydrogel is connected by carbon hydrogen oxide compounds [36]. In Figure 5f, the presence of copper in the DNDAH is evident, and it can be observed that the distribution of copper elements is relatively uniform. This indicates that the Cu_2_O in the primer could successfully release Cu^2+^ into the DNDAH coating through an electrochemical reaction. The working mechanism of Cu_2_O releasing Cu^2+^ into DNDAH to coordinate with AGM and Cu^2+^ releasing from DNDAH into seawater to play an anti-fouling role is shown Figure 5g.

#### 2.4.2. Anti-Protein Adsorption Ability

The anti-fouling characteristics of the DNDAH coating were analyzed via anti-protein-fouling using BSA as a protein model. After 1 day of immersion in BSA, more protein agglomeration was observed on the primer coating. The DNDAH coating exhibited a much better resistance, with an 88% reduction in protein attachment, compared to the coating without DNDAH, which exhibited a 74% resistance.

**Figure 6:**
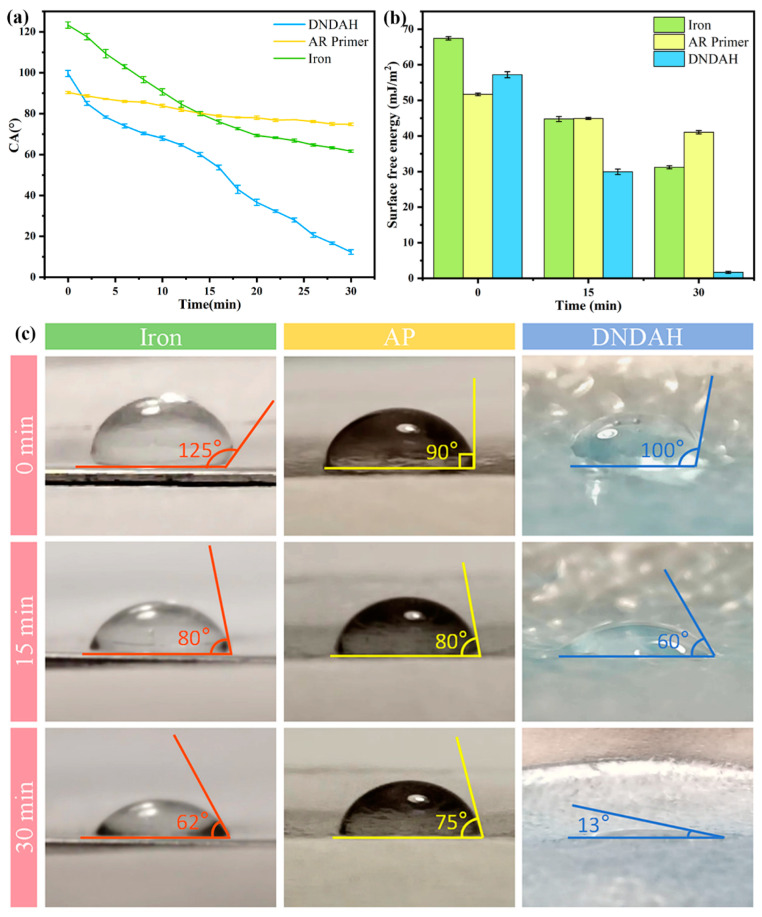
DNDAH anti-protein adsorption performance. (**a**) Contact angle changes on the surfaces of iron, AR primer, and DNDAH. (**b**) Surface energy changes on iron, AR primer, and DNDAH surfaces. (**c**) Water contact angle on iron, AP, and DNDAH at different times.

To comprehend the reasons behind the enhanced protein contamination resistance of the hydrogel coating, we conducted measurements of the DNDAH, the AR primer, and the iron wettability and surface free energy [37]. Figure 6a illustrates the dynamic water contact angle on the DNDAH, the AR primer, and iron over 30 min, and the pictures of the contact angle are shown in Figure 6c.

Initially, the DNDAH coating exhibited hydrophobic characteristics, with a high contact angle value of 100°. However, as time progressed, the contact angle dropped to 13°, indicating a transition from hydrophobic to super-hydrophilic. However, the contact angle of water on the AR primer and iron surfaces did not change significantly, and the small change in the angle was speculated to be due to the influence of gravity.

We counted the surface free energy (mJ/m^2^) of the three surfaces. Figure 6b demonstrates that the values of the iron and the AR primer both maintained high levels over 30 min, even if the rates of the iron went through a more obvious decrease; however, the value of the DNDAH dropped to nearly zero.

This transition was attributed to the migration of hydrophilic polymer chains from the interior to the surface of the hydrogel coating, forming a hydration layer which acted as an effective barrier against contact with proteins and other substances [19]. Previous findings have indicated that surfaces with anti-protein adsorption abilities generally exhibit characteristics of hydrophilicity and electrical neutrality. The surface of the hydrogel coating in this study met these requirements, demonstrating an excellent anti-protein adsorption performance. In contrast, the primer sample without the hydrogel coating was hydrophobic, with a significantly higher surface free energy compared to the DNDAH coating. The former was prone to surface-charging underwater, leading to an inferior anti-protein adsorption performance.

#### 2.4.3. Marine Simulation Experiment

**Figure 7:**
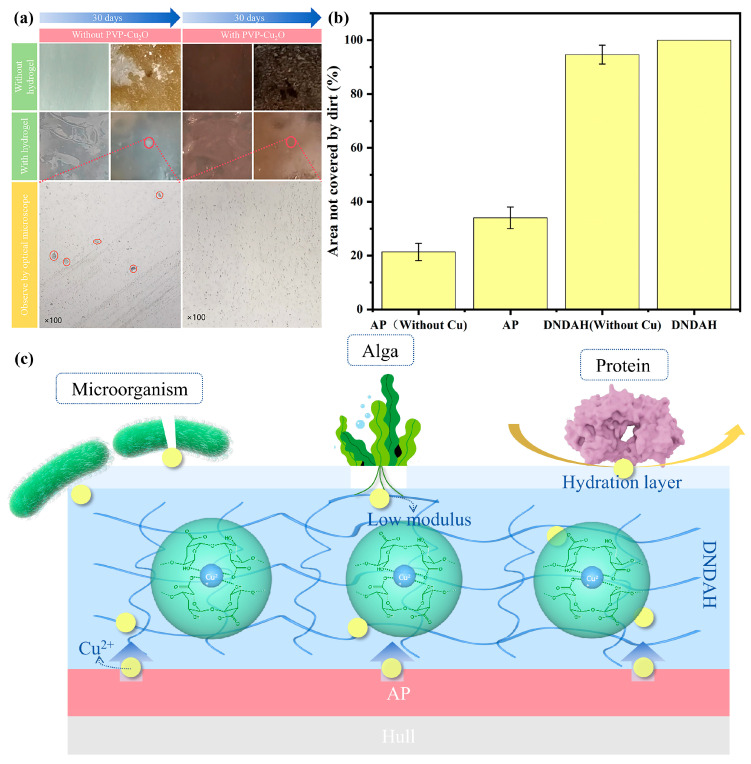
Ocean simulation experiment results. (**a**) Images of the coated panels before and after immersion in simulated marine environments. (**b**) The red circle in the picture shows the microorganisms attached to the coating. (**c**) DNDAH coating’s anti-microbial, algae, and protein adhesion mechanisms.

In order to test the long-term anti-biological adsorption performance of the A-H DL coatings in real marine environments, we simulated a seawater environment in a fish tank and immersed four types of coatings into it for 30 days. The microbial attachment on the samples from the fish tank after a 30-day-long immersion in seawater is illustrated in Figure 7a, while Figure 7b presents the ratio of untouched coating areas on four different coatings. The AR primer samples, which lacked the DNDAH coating, indicated a substantial microbial attachment, whereas the DNDAH coating revealed a minimal adhesion of microorganisms. This evidence validated the greater anti-microbial adsorption efficacy of the DNDAH coating compared to traditional marine coatings, hereby represented by the AR primer.

Despite the difficulty in discerning the differences in the anti-microbial adsorption performance of the two A-H DL coatings using the naked eye, thin slices of the hydrogel surface of each coating were observed under an optical microscope. As highlighted in Figure 7a, the DNDAH coating not anchored with the PVP-Cu_2_O AR primer showed a minimal microbial attachment of about 700 cells cm^−2^, as indicated by the red circle in the image. In stark contrast, the hydrogel coating anchored with the PVP-Cu_2_O AR primer demonstrated no microbial attachment. This observation verified that the Cu^2+^ released by the AR primer into the hydrogel fortified the DNDAH coating’s anti-biosorption ability. Previous studies have established the antibacterial tendencies of Cu^2+^. Hence, in an environment housing Cu^2+^, organisms’ survival rates dip significantly. Consequently, the DNDAH coating was said to employ its inherent hydrophilicity to resist biological attachment after the Cu_2_O in the AR primer released Cu^2+^ into the hydrogel coating by way of an electrochemical reaction. The experimental results demonstrated that A-H DL had a good anti-fouling performance over 30 days in seawater [19], which was sufficient to prove that it could withstand harsh marine environments over an extended period without losing its anti-fouling properties.

The mechanism of resisting biological adherence is illustrated in Figure 7c. Firstly, the surface of the hydrogel was not fit for microorganisms to live on, and, once a microorganism adhered to it accidentally, the Cu^2+^ in the gel would kill it immediately. For the algae in the sea, the hydrogel had a low modulus, thus the roots of algae could not grow on the surface easily. As for the proteins which generally come from mussels, the hydration layer formed on the surface of the hydrogel in the sea became a barrier to prevent these proteins from adhering to the ship. Such a process not only resisted biological adherence but also disrupted the physiological behavior of organisms that accidentally adhered to the coating through the Cu^2+^ present in the DNDAH coating. This feature allowed for further resistance to biological adsorption.

Briefly, the A-H DL coating has long-term anti-biofouling properties in real marine environments based on the surface characteristics of the hydrogel and the existence of Cu^2+^, and it has significant advantages in terms of its anti-biofouling properties compared to traditional ship coatings.

### 2.5. Considerations about Monitoring and Maintenance Strategies

The conditions in a real sea environment are very complex, so the functional lifespan of the A-H DL coating is unsure. Therefore, it is necessary to construct a mechanism to monitor the protective performance of the A-H DL coating and invent a convenient method to maintain it [38,39]. We propose two monitoring strategies here. The first is morphological observation, which is the traditional method of checking for mechanical damage to a coating. If there is some damage on the DNDAH coating, based on the reversible structure of the hydrogel system, workers could maintain the damage by filling the hydrogel precursor directly in the damaged part. The second method is sample testing. There are two aspects to this test, which overall involves sampling from the surface of the ship, including the coatings and any attached organisms. During this test, researchers first detect the Cu^2+^ content in the DNDAH to assess its current anti-biofouling performance. Second, the samples are sent to the laboratory for a microbiological analysis to determine which types of microorganisms have begun to exceed the prevention and control standards. If the lifespan of A-H DL is over, the whole coating, including the AR primer and the DNDAH, should be updated. In conclusion, in practical application scenarios, workers should consider two problems of this coating—appearance integrity and functional effectiveness—and should apply local or global maintenance according to the specific situations.

## 3. Conclusions

The present study successfully addressed the limitations of traditional marine anti-fouling coatings by developing the innovative A-H DL coating system. This dual-layer design, comprising an AR primer with controlled Cu^2+^ release and a DNDAH coating with robust anchoring and self-lubricating properties, demonstrated exceptional performance in marine environments.

Extensive testing revealed the A-H DL coating’s superior anti-biofouling capabilities. After 30 days of immersion, the coating exhibited remarkable stability, with no corrosion or microbial attachment. The uniform copper distribution, the impressive protein adsorption resistance, and the significantly reduced microbial attachment confirmed the effectiveness of the system’s anti-fouling mechanisms.

In conclusion, the A-H DL coating presents a promising solution for overcoming the challenges of marine anti-fouling. This coating focuses on the problems of environmental friendliness and a short service life in traditional ship coatings, and, by combining these with the characteristics of various new anti-biofouling coatings, it achieves a long-term underwater anti-biofouling performance while reducing pollution of the oceans. Its unique design, superior performance, and potential for environmental benefits make it a valuable addition to the arsenal of marine technologies aimed at protecting our oceans and ensuring the sustainable operation of marine infrastructure. Despite the promising laboratory results, the potential environmental impacts of the copper released by the coating require comprehensive evaluation. Further research and development are also essential to enhancing the A-H DL system and exploring its wider applications across various marine scenarios.

Developing a new coating entails various challenges that warrant consideration. For instance, scaling-up experiments may pose construction issues, longer-term durability testing is required, and a comprehensive environmental assessment involving systematic scientific approaches is essential. To address these limitations and refine the A-H DL coating system, future research could focus on optimizing the application methods to overcome scalability issues, conducting extended durability tests under varying environmental conditions, and performing comprehensive environmental impact assessments. Additionally, exploring the underlying mechanisms driving this coating’s anti-biofouling properties through theoretical frameworks or computational modeling could provide valuable insights. Moreover, investigating the integration of novel materials or additives to enhance efficacy and environmental sustainability would further refine the A-H DL coating system for broader applicability in marine environments.

## 4. Materials and Methods

### 4.1. Materials

All the starting materials and solvents are commercially available and were applied without further purification. The following materials were used to prepare the AR primer. Polyvinylpyrrolidone (PVP, K30) was acquired from Minerui Chemical Technology (Shanghai, China). NaOH, Na_2_SO_3_, Na_2_SO_4_, and CuSO_4_ were supplied by Kaitong Chemical Reagent (Tianjin, China). 3-(trimethoxymethylsilyl) propyl methacrylate (TMSPMA) was acquired from D&B Biological Science and Technology (Shanghai, China). Nano-SiO_2_ was acquired from Shanghai Maclean Biochemical Technology Co., Ltd. (Shanghai, China). Methyl methacrylate (MMA), butyl acrylate, hydroxyethyl acrylate, acrylic acid, styrene, azodiisobutyronitrile, *tert*-butyl benzoate peroxide, dodecyl mercaptan, *N*, *N*-dimethylethanolamine, ethylene glycol butyl ether, and isopropanol were supplied by Aladdin Biochemical Technology Co., Ltd. (Shanghai, China). Anhydrous ethanol was acquired from Fuyu Fine Chemical Co., Ltd. (Tianjin, China). Anti-foam agent 024 was obtained from BYK-CHEMIE Co., Ltd. (Shanghai, China).

The following materials were used to prepare and characterize DNDAH. 3-aminopropyltriethoxysilane (APES) was acquired from Tokyo Chemical Industry Shanghai Huacheng Industrial Development Co., Ltd. (Shanghai, China). 3-ethylcarbodiimide hydrochloride, *N*-hydroxysuccinimide, alginate, ammonium persulfate (APS), acrylamide (AAm), *N*, *N*, *N’*, *N’*-Tetramethylethylenediamine (TEMED), and Coomassie brilliant blue were acquired from Rhawn Co., Ltd. (Shanghai, China). Bovine serum albumin (BSA) was purchased from Sinopharm Chemical Reagent Co., Ltd. (Beijing, China). NaCl, MgSO_4_, CaCl_2_, NaHCO_3_, and KCl were acquired from Aladdin Biochemical Technology Co., Ltd. (Shanghai, China). (The above-mentioned drugs were administered in minimal amounts, posing no significant safety risks related to the apparatus. The standard safety precautions implemented during the experiments were adequate to ensure the overall safety of the procedures.)

### 4.2. Preparation of AR Primer

The first step was preparing an AR primer that was composed of PVP-Cu_2_O microcapsules, TMSPMA-SiO_2_, waterborne AR, and other auxiliaries.

Synthesis of PVP-Cu_2_O microcapsules. Under certain conditions, Na_2_SO_3_ can reduce Cu^2+^ to Cu^+^ and then generate Cu_2_O particles. In this process, a growth center was provided by the PVP macromolecule for the Cu_2_O crystal to precipitate from the solution and coated the surface of the Cu_2_O particles to form microcapsules under the action of coagulant (Na_2_SO_4_) [5,35]. The possible chemical reactions that may occur during this process are explained below.

We dissolved 25 g of CuSO_4_·5H_2_O and 2.5 g of PVP in 150 mL of water, and this system was transferred into a 500 mL three-necked flask with a thermometer and a stirrer. We heated the system to 70 °C and maintained heating with stirring at 250 rpm. We added 10 mL of 20% NaOH solution over 10 min. After that, we added 6.0 mL of Na_2_SO_3_ solution (25%) at once and allowed it to react for 20 min. Then, we added 10.0 mL of Na_2_SO_4_ solution (25%) over 10 min and allowed it to react for 0.5–1 h. Upon completion of the reaction, we filtered the resulting solution, washed the filter cake twice with anhydrous ethanol and deionized water, and dried it at 45 °C for 3 h. Finally, we ground the product to obtain powdered microcapsules and weighed them.

Synthesis of TMSPMA-SiO_2._ We took 0.85 g of TMSPMA dissolved in 4.0 mL water, adjusting the pH value to 3 with acetic acid. Afterwards, we weighed 2.0 g of SiO_2_, dissolved it in a 1:1 (*v*/*v*) solution of 200.0 mL ethanol and water, and allowed it to ultrasonically disperse for 30 min. Then, we mixed the dispersion with the solution prepared in the first step and heated it in a water bath at 60 °C for 4 h. After the reaction had been completed, we took the product and centrifuged it at 8000 rpm for 10 min [40]. Finally, the separated SiO_2_ was dried in an oven for 24 h at 45 °C, resulting in the attainment of TMSPMA-SiO_2_.

Synthesis of waterborne AR. A low-toxicity, highly soluble solvent solution was used for polymerization, facilitating the transport of the monomer mixture solution into the solvent through the “starvation” dropwise addition method [41]. In brief, we heated a mixture of 5.0 mL isopropyl alcohol and 5.0 mL ethylene glycol butyl ether in a three-necked flask using a water bath until it reached 85 °C. Following this, we combined 8.3 mL of butyl acrylate, 3.3 mL of hydroxyethyl acrylate, 1.7 mL of acrylic acid, 0.3 mL of *n*-dodecyl mercaptan, 10.0 mL of methyl methacrylate, 3.3 mL of styrene, and 0.4 g of azodiisobutyronitrile. We mixed and dispersed this combination in a beaker for 30 min. We transferred the mixed solution into a constant-pressure funnel, adjusted the flow rate, and gradually introduced it into the system of step 1 over 2 h. Upon completion of the reaction, we cooled the system to 55 °C. We added 20.0 mL *N*, *N*-dimethylethanolamine to the constant-pressure funnel and slowly incorporated it drop by drop, stirring for 10 min. Subsequently, we poured the resulting product into the beaker until further use.

Preparation of the AR primer. Painting was performed by incorporating 0.67 g TMSPMA-SiO_2_, relative to the total paint content, into 20 mL of amino resin, stirring thoroughly [42,43,44]. Subsequently, we added 0.335 g PVP-Cu_2_O and stirred the mixture again [5,35,45]. Following this, we introduced the previously prepared 40 mL of AR, as per the experimental steps mentioned earlier, into the amino resin and stirred the combination. We transferred the blended solution into a material bucket. Then, we poured it into the material bucket. We kept the disperser at a speed of 1 500 rpm, sequentially dispensing the materials into the barrel. We added 1 mL of defoamer, 2 mL of co-solvent (xylene), and 1 mL of catalyst. We mixed 1 mL of leveling agent and 2 mL of dispersant for an additional 15 min to achieve the final primer [35,42,46,47].

We cleaned the iron sheet sequentially with distilled water and methanol, followed by sanding with sandpaper and a subsequent step of methanol cleaning. We utilized a spray gun to apply the acrylic coating prepared according to the aforementioned steps onto the thoroughly cleaned iron sheet [44]. Subsequently, we placed the sprayed iron sheet in an oven, initially set the oven temperature to 60 °C, and adjusted it to 80 °C when it reached 60 °C. Once the temperature reached 80 °C, we maintained it for 15 min. We adjusted the oven temperature to 150 °C, and, after it reached this temperature, we kept it constant for 30 min to complete the curing process, resulting in the formation of the AR primer coating.

### 4.3. Preparation of A-H DL

Second, to prepare the A-H DL, we coated the hydrogel precursor of DNDAH on the AR primer, and the detailed content of this step is as follows.

Preparation of alginate gel microspheres (AGM) grafted with anchoring groups. The amino group in 3-aminopropyltriethoxysilane can undergo condensation with the carboxyl group in the long chain of alginate, resulting in the formation of an alginate polymer-grafted hydrogel with an anchor group (–Si (CH_3_)). Incorporating this polymer into the gel coating system allowed for the creation of a second gel network, providing additional anchoring for the primer.

We dissolved 4.0 g of sodium alginate in water, then added 1.4 mL of APES, 7.6 g of 3-ethylcarbodiimide hydrochloride, and 0.7 g of *N*-hydroxysuccinimide, stirring continuously for 24 h [48,49] to form a gel-like polymer. We transferred the obtained polymer gel into a dialysis bag (MW: 140,000) for a 3-day dialysis process. Subsequently, we dried the polymer in an oven for 48 h at a temperature of 45 °C. The dried polymer was then subjected to grinding in a ball mill, resulting in micron-sized gel microspheres, obtaining AGM grafted with anchoring groups.

**Figure 8:**
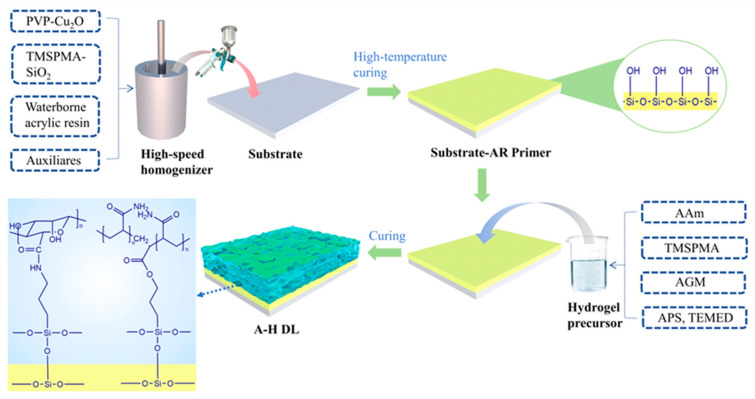
A-H DL coating production process.

Preparation of A-H DL coating. Figure 8 shows the production process of the A-H DL coating. The copolymerization of AAm and TMSPMA resulted in the formation of the first network of hydrogels, establishing the initial anchoring with the primer [16,20,24]. Incorporating the gel microspheres, prepared as per the aforementioned steps, into the gel precursor enabled the creation of a second network of hydrogels. This occurred through coordination cross-linking with the Cu^2+^ released from the primer, and it combined with the primer to form the second anchoring [35]. The first and second networks could be integrated through the condensation of a coupling agent, ultimately forming a hydrogel coating.

In 10 mL distilled water, we introduced 0.1 g of AGM and ultrasonically dispersed the mixture for 30 min to create a gel microsphere suspension. We added 1.0 g of AAm and 150 μL of TMSPMA into the suspension, stirring for 10 min. Subsequently, we introduced 200 μL of APS water solution (0.27 mol L^−1^) and stirred for 10 min, following this with the addition of 20 μL of TEMED, stirring for 1 min [22,23,50,51]. 

We immediately applied the resulting mixture onto the primer coating prepared following the steps mentioned earlier. We allowed it to stand at 35 °C for 30 min to form a hydrogel, thereby obtaining a dissipative dual-network dual-anchoring hydrogel coating anchored to the primer coating.

### 4.4. Characterization

For the hydrogel coating, FTIR (Nicolet IS50, Shanghai, China) spectroscopy was used to determine its chemical composition. After that, the surface morphology of the hydrogel and the morphology and distribution of the gel microspheres were observed by SEM (EM-30plus, Beijing, China). In the case of the primer coating, Fourier transform infrared spectroscopy was used to characterize the dispersed nano-PVP-Cu_2_O microcapsules and coupling agent-modified nano-silica within the primer, confirming their chemical composition. Further validation of the chemical composition of microcapsules was conducted through XRD (X Pert3 Power, PANalytical B.V., Beijing, China). Additionally, the morphological characteristics and the particle size of two types of nano-particles were observed through TEM (13004511JEM-2100, Beijing, China).

### 4.5. Mechanical Performance Testing 

Tensile performance testing. The tensile properties of the AAm single-network hydrogel (SNSAH) and DNDAH were tested with a general testing machine (MAW-600B, Beijing, China) under the condition of water swelling equilibrium. Two hydrogels were cut into dog bone shapes (gauge length: 12 mm; width: 2 mm), and the sample was stretched along the length direction at an extension velocity of 100 mm min^−1^ [52]. We recorded the critical fracture tension and calculated the tensile strength using the following formula:

(1) $$\mathrm{Tensile}\;\mathrm{strength}=\frac{\mathrm{Maximum}\;\mathrm{tensile}\;\mathrm{force}}{\mathrm{Original}\;\mathrm{cross}\;-\;\mathrm{sectional}\;\mathrm{area}}$$

Adhesion properties’ experiments. The first section of the experiments was a cross-cut test to prove the excellent adhesion property between AR primer and substrate [44]. We used the knife to draw two lines on the prepared AR primer and then drew two lines in the vertical direction that intersected with the previous line to obtain a grid. After that, we applied tape to the marked area and lightly pressed to ensure good contact between the tape and the coating, then peeled off the tape at a certain angle and speed, and observed if the coating peeled off with the tape. We observed whether the paint film had been peeled off from the substrate by the adhesive tape in order to determine the adhesion performance of the AR primer [53]. Second, we proved the anchoring performance between the DNDAH and the AR primer. We used the spring dynamometer to slowly pull the hydrogel coating upward at a constant speed to peel it off the AP, and we recorded the spring dynamometer readings every 2 s [50]. We prepared three types of samples: the first one had a paint coat composed of the primer without TMSPMA-SiO_2_, combined with DNDAH; the second one had a paint coat composed of the primer with TMSPMA-SiO_2_, combined with SNSAH; and the third one had a paint coat composed of the primer with TMSPMA-SiO_2_, combined with DNDAH. We submerged the aforementioned samples in water for 30 days and evaluated their adhesion to the primer.

### 4.6. Cu*^2+^* Release and Functional Testing

Cu^2+^ release detection. We conducted a Cu^2+^ release experiment in artificial seawater for 30 days on substrates coated with the AR primer and the A-H DL coating, respectively. In alkaline solutions, Cu^2+^ can react with a copper reagent to form a yellow colloidal complex, which has the highest absorption at 450 nm and can maintain stability for about 20 min. Therefore, the concentration of Cu^2+^ can be determined by measuring the absorbance and standard curve using a spectrophotometer, and the release rate of copper ions can be calculated using the following formula:

(2) $$R=\frac{c\times V\times 24\;h}{S\times t}$$

where *R* is the copper ion release rate (μg·cm^−2^·d^−1^), *c* is the measured Cu^2+^ concentration (μg/mL), *V* is the volume of artificial seawater (mL), *S* is the area of the coating template (cm^2^), and t is the testing time (h) [35]. On this basis, the Cu^2+^ release rate of the two types of coating samples in artificial seawater was measured using spectrophotometry (TU-1901, Beijing, China), and the release performance of the coatings was characterized. We immersed the DNDAH coating, adhered to the primer with PVP-Cu_2_O, in water for 30 days [54], followed by the freeze-drying of the hydrogel coating. The distribution and proportion of the elements in the gel coating after the release of Cu^2+^ into the hydrogel were examined using EDS spectroscopy (JSM-7500 F, JEOL, Tokyo, Japan).

Anti-protein adsorption experiment. At room temperature, we dissolved 20 mg of BSA in 400 mL of phosphate-buffered solution (PBS, pH = 7.2) to obtain 0.05 mg mL^−1^ BSA solution. We placed 30 mL of PBS in a Petri dish (d = 48 10 cm) and prewetted the hydrogel sample in it for 2 h [55]. Subsequently, we removed the PBS from the culture dish, added 30 mL of the BSA solution, and floated the hydrogel sample in BSA, immersing it on the side with the hydrogel coating [56].

After 24 h, we rinsed the protein that did not adhere to the hydrogel coating surface with 10 mL of PBS to dislodge it. We combined the PBS rinsing solution and BSA immersion solution, and 20 mL of PBS was added to the mixture, kept it standing for 2 min, thus obtaining test solution A. The BSA content in test solution A was detected according to Bradford [57] to determine the anti-protein adhesion effect on the hydrogel coating.

Briefly, we mixed 1.00 mL of protein solution to be tested with 10.0 mL Coomassie brilliant blue working solution. After 2 min, we measured the absorbance of the mixture solution using UV spectrophotometry (TU-1901, Beijing, China) at 595 nm for 1 h. Then, we calculated the BSA concentration using a standard curve. We replaced the hydrogel sample with the primer sample without the hydrogel coating and repeated the above experimental operation.

The anti-protein adsorption rate (*A*) was calculated through the following formula, which is expressed, by comparison, with the BSA concentration [19]:

(3) $$A=\frac{c_{1}}{c_{0}}\times 100\%$$

where *c*_0_ is the concentration of BSA solution which has not been used, and *c*_1_ is the protein concentration without adhesion on the coating. In the preparation of all solutions, bubbles must be avoided, or else the experimental results would be affected.

Hydrophobicity test of hydrogel coating surface. We placed the prepared DNDAH, AP, and iron coating in the open air, applied a drop of water onto its surface [19], and measured the contact angle of the water droplet every 2 min. Then, we calculated the surface energy of three kinds of coatings underwater according to the following formula [58]:

(4) $$\gamma_{sl}=\frac{\gamma_{lg}}{2}\left(\sqrt{1+{sin}^{2}}\theta -cos\theta \right)0\leq \theta \leq 180^{\circ }.$$

*γ_sl_* is the surface tension between solid and liquid; *γ_lg_* is the surface tension between gas and liquid; ϴ is the contact angle.

Marine simulation experiment. We introduced artificial seawater (NaCl: 3.0 wt%; MgSO_4_: 0.32 wt%; CaCl_2_: 0.12 wt%; NaHCO_3_: 0.02 wt%; and KCl: 0.072 wt% [59]) into a fish tank measuring 30 × 20 × 30 cm. We added several common seaweeds and three zebrafish to simulate an ocean-like ecological environment [23,45]. We created four types of samples: The first had a paint coat consisting of the primer without PVP-Cu_2_O, combined with the DNDAH coating. The second had a paint coat comprising the primer with PVP-Cu_2_O, combined with the DNDAH coating. The remaining two samples were primer coatings not combined with the DNDAH coating, one with added PVP-Cu_2_O and the other without PVP-Cu_2_O. We submerged the aforementioned samples in the fish tank and retrieved them after 30 days [45]. We extracted the gel coating samples and placed them under an optical microscope to observe plankton attachment on the coating surface.

### 4.7. Statistical Analysis

We employed statistical methods to compare group differences and evaluate their statistical significance. We utilized the mean standard deviation for the analysis of the data collected from distinct parallel surveys. We performed a statistical analysis using the Office and Origin software. The experimental results of mechanistic reasoning were explained and analyzed by drawing schematic diagrams using C4D, Office, and Photoshop. The significance level was set to * *p* < 0.05, indicating statistical significance; ** *p* < 0.01 denoted a high statistical significance; *** *p* < 0.001 signified the highest level of statistical significance [60].

## Data Availability

The original contributions presented in the study are included in the article, further inquiries can be directed to the corresponding authors.
